# *BRAF* V600E potentially determines “Oncological Resectability” for “Technically Resectable” colorectal liver metastases

**DOI:** 10.1002/cam4.4227

**Published:** 2021-09-18

**Authors:** Shin Kobayashi, Shinichiro Takahashi, Shogo Nomura, Motohiro Kojima, Masashi Kudo, Motokazu Sugimoto, Masaru Konishi, Naoto Gotohda, Hiroya Taniguchi, Takayuki Yoshino

**Affiliations:** ^1^ Department of Hepatobiliary and Pancreatic Surgery National Cancer Center Hospital East Kashiwa Japan; ^2^ Department of Gastroenterology and Gastrointestinal Oncology National Cancer Center Hospital East Kashiwa Japan; ^3^ Clinical Research Support Office National Cancer Center Hospital East Kashiwa Japan; ^4^ Division of Pathology Research Center for Innovative Oncology National Cancer Center Hospital East Kashiwa Japan

**Keywords:** *BRAF* V600E, colorectal liver metastases, hepatectomy, resectable, surgery

## Abstract

Despite reports on poor survival outcomes after hepatectomy for colorectal liver metastases (CRLM) with *BRAF* V600E mutation (m*BRAF*) exist, the role of m*BRAF* testing for technically resectable cases remains unclear. A single‐center retrospective study was performed to investigate the survival outcomes of patients who underwent upfront hepatectomy for solitary resectable CRLM with m*BRAF* between January 2005 and December 2017 and to compare them with those of unresectable cases with m*BRAF*. Of 172 patients who underwent initial hepatectomy for solitary resectable CRLM, m*BRAF*, *RAS* mutations (m*RAS*), and wild‐type *RAS*/*BRAF* (wt*RAS*/*BRAF*) were observed in 5 (2.9%), 73 (42.4%), and 93 (54.7%) patients, respectively. With a median follow‐up period of 72.8 months, *mBRAF* was associated with a significantly shorter OS (median, 14.4 months) than wt*RAS*/*BRAF* (median, not reached [NR]) (hazard ratio [HR], 27.6; *p* < 0.001) and m*RAS* (median, NR) (HR, 9.9; *p* < 0.001), and m*BRAF* had the highest HR among all the indicators in the multivariable analysis (HR, 17.0; *p* < 0.001). The median OS after upfront hepatectomy for CRLM with m*BRAF* was identical to that of 28 unresectable CRLM with m*BRAF* that were treated with systemic chemotherapy (median, 17.2 months) (HR, 0.78; *p* = 0.65). When technically resectable CRLM are complicated with m*BRAF*, its survival outcome becomes as poor as unresectable cases; therefore, those with m*BRAF* should be considered as oncologically unresectable. Patients with CRLM should undergo pre‐treatment m*BRAF* testing regardless of technical resectability.

**Clinical trial registration number:** UMIN000034557.

## Graphical abstract

A single‐center retrospective study was performed to investigate the survival outcomes of patients who underwent upfront hepatectomy for solitary resectable CRLM with BRAF V600E mutation between January 2005 and December 2017 and to compare them with those of unresectable cases with the mutation. When technically resectable CRLM are complicated with BRAF V600E, its survival outcome becomes as poor as unresectable cases; therefore, those with the mutation should be considered as oncologically unresectable.

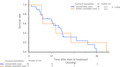

## 1 INTRODUCTION

Colorectal cancer (CRC) is the third most common cancer and the second leading cause of cancer‐related deaths worldwide.^1^ The liver is the most common site of metastatic CRC (mCRC), with approximately 30% of patients with CRC developing liver metastases over the course of their disease.^2^ Since both the median recurrence‐free survival (RFS) and overall survival (OS) rates after hepatectomy for CRLM are high at approximately 20 and 60 months, respectively,^3^ hepatectomy is generally recommended for curative intent.^4^, ^5^ However, even apparently resectable CRLM are a heterogeneous disease in which approximately 70%–80% of patients eventually experience recurrence.^6^ Despite the definition of multiple CRLM ≥5 lesions as oncologically unresectable in the consensus guidelines by the European Society for Medical Oncology (ESMO),^5^ additional biomarkers to define the oncological resectability remain warranted.

The *RAS*–*RAF*–*MEK*–*ERK*–*MAP* kinase pathway regulates cellular growth and is activated in many human cancers.^7^ *BRAF* is one of the three *RAF* genes that code for serine/threonine kinases, and V600E is the most dominant type of its mutation in which disruption of the A‐loop and the P‐loop of the *BRAF* mutant protein directly phosphorylates downstream *MEK* activity and *BRAF* kinase activity increases up to 700‐fold of the wild type to stimulate rapid cell growth of the tumor.^8^ In today's era of personalized precision medicine, the evaluation of *RAS*/*BRAF* V600E mutations at the time of diagnosis of unresectable mCRC has been strongly recommended in clinical practice guidelines due to their prognostic significance and their utility in guiding the selection of optimal chemotherapeutic regimens.^4^, ^5^ For resectable CRLM, *RAS*/*BRAF* mutations are associated with poor prognosis, with *BRAF* V600E mutation (m*BRAF*) as a poorer prognostic factor for OS.^9^, ^10^, ^11^, ^12^, ^13^ When resectable CRLM were complicated with m*BRAF*, OS after hepatectomy for CRLM was reported to be 22–31 months, while that of unresectable mCRC treated with systemic chemotherapy was reported to be 19 months.^14^ Although m*BRAF* is recognized as negatively associated with poor survival after hepatectomy, the role of m*BRAF* testing before hepatectomy remains unclear due to the absence of studies that directly compare the survival outcome of patients treated with hepatectomy with those treated with systemic chemotherapy.

The purpose of this study was to investigate the survival outcomes of surgical patients who underwent upfront hepatectomy for CRLM without neoadjuvant chemotherapy depending on *RAS*/*BRAF* mutational status, to compare the survival outcomes of patients with *BRAF* V600E‐mutant CRLM depending on technical resectability, and to clearly define the role of the m*BRAF* testing before hepatectomy for CRLM. This study focused solely on patients who had undergone hepatectomy for solitary resectable CRLM without neoadjuvant chemotherapy in order to clarify the impact of m*BRAF* on survival outcomes and to elucidate the natural history of patients with m*BRAF* after hepatectomy. We opted to focus on this clearly resectable cohort as other factors including the number of tumors, use of neoadjuvant chemotherapy, and initial resectability may act as confounding factors for the effect of m*BRAF*.

## 2 METHODS

### 2.1 Study design

Patients who were treated for CRLM between January 2005 and December 2017 at the National Cancer Center Hospital East, Kashiwa, Japan, were included in the study. In the first analysis, patients who underwent upfront hepatectomy without neoadjuvant chemotherapy for solitary resectable liver‐limited tumors at preoperative diagnosis were included. Inclusion criteria were those CRLM cases diagnosed preoperatively by ultrasound, computed tomography, and magnetic resonance imaging and histologically proven as metastatic adenocarcinoma of CRC origin. CRLM that required the resection of >70% of the entire liver, all three major hepatic veins, or bilateral branches of the hepatic artery/portal vein were considered technically unresectable and were excluded from the analysis. Since guidelines by the ESMO and the Japanese Society for Cancer of the Colon and Rectum have not yet established the efficacy and feasibility of neoadjuvant chemotherapy for technically resectable solitary CRLM,^5^, ^15^ neoadjuvant chemotherapy has not been offered to patients with solitary resectable CRLM in Japan. Survival outcomes including RFS, time to surgical failure (TSF), and OS after initial hepatectomy were analyzed in relation to *RAS*/*BRAF* V600E mutations and other clinicopathological factors.

In the second analysis, patients with m*BRAF* among those who received systemic chemotherapy for initially unresectable CRLM were included. OS after the start of the first‐line chemotherapy was analyzed and compared with that of patients who underwent upfront hepatectomy without neoadjuvant chemotherapy for *BRAF* V600E‐mutant solitary resectable liver‐limited tumors at preoperative diagnosis. Because of the retrospective nature of the study and the absence of invasive interventions, patients` personal written consents were waived. The study protocol was approved by the review board of the National Cancer Center Hospital East (approval number: 2018‐272) and was registered in the clinical trial registration system (UMIN000034557). This study received financial support from the National Cancer Center Research Development Fund (Research number: 30‐A‐8, Principal investigator: Shinichiro Takahashi).

### 2.2 Study outcomes

Standard clinicopathological data related to patient factors (age and sex), primary CRC factors (location, depth, lymph node metastases, pathology, and adjuvant chemotherapy after primary CRC resection), CRLM factors (timing, largest diameter, location, extrahepatic metastases status, preoperative chemotherapy for CRLM, adjuvant chemotherapy after hepatectomy, systemic chemotherapy regimens for unresectable tumors, CEA, carbohydrate antigen 19‐9 [CA19‐9], and residual tumor after hepatectomy), mismatch repair (MMR) protein status, microsatellite instability (MSI) status, and survival outcomes (RFS, TSF, and OS) along with information on tissue *RAS*/*BRAF* V600E mutations were retrospectively obtained. RFS was defined as the time from the date of hepatectomy to the date of the first radiological recurrence of the disease or to the date of death due to any cause. TSF was defined as either the time from the date of hepatectomy to the date of the radiological recurrence of unresectable disease or to the date of death due to any cause. OS was calculated either from the date of hepatectomy in the first analysis or the date of initiation of the first‐line chemotherapy in the second analysis until either the date of death due to any cause or the last follow‐up. Survival analysis was updated as of February 2021. As for the surveillance schedule, current Japanese guidelines recommend performing serial measurements of CEA and CA19‐9 levels and thoracoabdominal computed tomography scans every 3 and 6 months, respectively, after the resection of Stage I to III CRC.^15^ The same schedule or an even more intensive schedule is recommended after the resection of Stage IV CRC or recurrent metastases. Generally, patients in this study were followed up every 3 months.

### 2.3 *RAS* and *BRAF* V600E mutational analysis

DNA was extracted from formalin‐fixed paraffin‐embedded tissue specimens which were selected from CRLM, primary CRC, or metastatic lung tumor in 159, 12, and 1 patients, respectively, and mutational analysis of *RAS*/*BRAF* V600E was centrally performed using a multiplex PCR‐based mutation detection kit (MEBGEN RASKET Kit; Medical and Biological Laboratories). *RAS* mutations (m*RAS*) were defined as mutations in codons 12, 13, 59, 61, 117, or 146 of *KRAS* or *NRAS*. Wild‐type *RAS*/*BRAF* (wt*RAS*/*BRAF*) was defined as those cases in which both *RAS*/*BRAF* V600E mutations were absent. In patients with *BRAF* V600E mutation, MMR protein status was determined by subjecting tumor samples to immunohistochemical analyses. *RAS*/*BRAF* V600E testing was retrospectively performed for this study and did not affect the clinical judgment of perioperative treatment in the first analysis. Meanwhile, it was performed as a part of clinical practice to decide chemotherapy regimens in the second analysis.

### 2.4 Statistical analyses

Statistical analyses of categorical variables were performed using the chi‐squared test or Fisher's exact test. Analyses of numerical variables were performed using the Mann–Whitney test. Survival curves were estimated and compared using the Kaplan–Meier method and the log‐rank test. Univariable and multivariable risk analyses were performed using Cox proportional hazards regression analysis. All *p* values are reported as two‐sided values, and *p* values < 0.05 were considered statistically significant. All statistical analyses were performed using the software EZR (ver. 1.37).^16^

## 3 RESULTS

### 3.1 Clinicopathological characteristics

**FIGURE 1:**
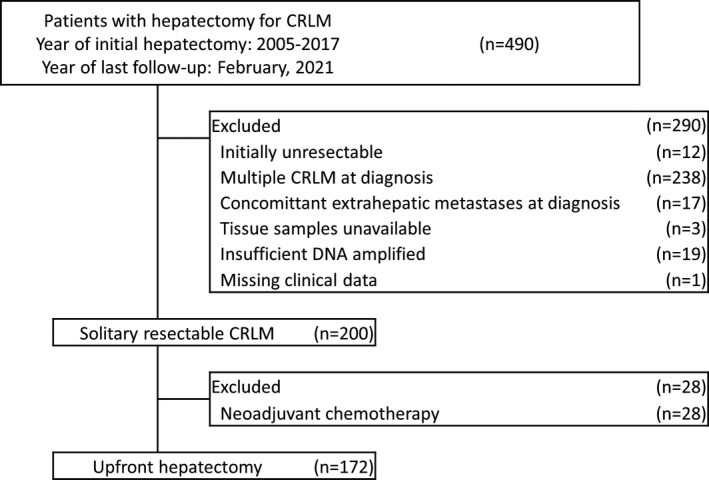
Consort diagram of eligible patients

In the first analysis, 172 patients who underwent upfront hepatectomy for solitary resectable CRLM at preoperative diagnosis without neoadjuvant chemotherapy were eligible for this study (Figure 1). m*BRAF*, m*RAS*, and wt*RAS*/*BRAF* were identified in 5 (2.9%), 73 (42.4%), and 93 (54.7%) patients, respectively. Additionally, details of *KRAS* mutations were as follows: codon 12, 52 (30.2%); codon 13, 5 (2.9%); codon 59, 1 (0.6%); codon 117, 1 (0.6%); codon 146, 5 (2.9%); unspecified, 1 (0.6%). Also, details of *NRAS* mutations were codon 12 in 4 (2.3%) and codon 61 in 3 (1.7%). No preoperative baseline characteristics were significantly correlated with mutational status except for the pre‐hepatectomy CA19‐9 level, which was significantly higher in patients with m*BRAF* than in those with m*RAS* or wt*RAS*/*BRAF* (median, 137.2 IU/ml vs. 17.3 IU/ml vs. 14.2 IU/ml; *p* = 0.03; Table 1). Incidental peritoneal metastases were intraoperatively identified in one patient with m*BRAF* (20.0%), none among those with m*RAS* (0%), and two among those with wt*RAS*/*BRAF* (2.1%; *p* = 0.04). Meanwhile, incidental multiple CRLM were identified in four patients among those with m*RAS* (5.5%), one among those with wt*RAS*/*BRAF* (1.1%), and none among those with m*BRAF* (0%; *p* = 0.28). The extent of hepatectomy was not different among the three groups and all lesions including incidental ones were macroscopically completely resected.

**TABLE 1:** Baseline characteristics

Factor	Group	Genomic mutational status			
		Wild‐type *RAS*/*BRAF*	*RAS* mutations	*BRAF* V600E mutation	
		*n* = 94	*n* = 73	*n* = 5	*p* value
**Patient factor**					
Sex, No. (%)	Female	29 (30.9)	34 (46.6)	2 (40.0)	0.12
	Male	65 (69.1)	39 (53.4)	3 (60.0)	
Age at hepatectomy, median [range]	Years	67 [32, 84]	67 [27, 87]	71 [43, 76]	0.64
Primary colorectal tumor factor					
Location, No. (%)	Right‐sided	13 (13.8)	18 (24.7)	2 (40.0)	0.10
	Left‐sided	81 (86.2)	55 (75.3)	3 (60.0)	
Depth of invasion, No. (%)	T1–3	73 (77.7)	55 (77.5)	4 (80.0)	0.99
	T4	21 (22.3)	16 (22.5)	1 (20.0)	
Lymph node metastases, No. (%)	Absent	44 (46.8)	28 (38.9)	0 (0.0)	0.09
	Present	50 (53.2)	44 (61.1)	5 (100.0)	
Pathology of the primary tumor, No. (%)	Well diff. adenocarcinoma	26 (27.7)	19 (26.0)	1 (20.0)	0.92
	Others	68 (72.3)	54 (74.0)	4 (80.0)	
Adjuvant chemotherapy after original tumor resection, No. (%)	No	21 (58.3)	18 (62.1)	2 (100.0)	0.50
	Yes	15 (41.7)	11 (37.9)	0 (0.0)	
CRLM factors					
Timing of CRLM, No. (%)	Synchronous	33 (35.1)	25 (34.2)	2 (40.0)	0.50
	Early metachronous <1 year	27 (28.7)	22 (30.1)	3 (60.0)	
	Late metachronous ≥1 year	34 (36.2)	26 (35.6)	0 (0.0)	
Diameter of CRLM at diagnosis, median [range]	mm	26 [4, 80]	23 [8, 68]	19 [11, 46]	0.13
CEA at diagnosis, median [range]	ng/ml	7.3 [1.0, 417.8]	8.6 [1.0, 2165.0]	4.0 [3.0, 93.3]	0.72
CA19‐9 at diagnosis, median [range]	IU/ml	14.0 [0.6, 3710.0]	17.3 [0.1, 1432.0]	137.2 [10.2, 563.1]	0.03
Extent of hepatectomy^a^	<1 sectorectomy	76 (80.9)	58 (79.5)	3 (60.0)	0.395
	1 sectorectomy	10 (10.6)	10 (13.7)	2 (40.0)	
	≥2 sectorectomy	8 (8.5)	5 (6.8)	0 (0.0)	
Residual tumor after hepatectomy, No. (%)	R0	90 (95.7)	69 (94.5)	4 (80.0)	0.30
	R1	4 (4.3)	4 (5.5)	1 (20.0)	
Adjuvant chemotherapy, No. (%)	No	55 (58.5)	36 (49.3)	2 (40.0)	0.41
	Yes	39 (41.5)	37 (50.7)	3 (60.0)	

Abbreviations: CA19‐9, carbohydrate antigen 19–9; CEA, carcinoembryonic antigen; CRLM, colorectal liver metastases.

^a^ The extent of hepatectomy was defined according to the Couinaud's sector.

### 3.2 Survival analysis after hepatectomy according to *RAS*/*BRAF* V600E mutational status

**FIGURE 2:**
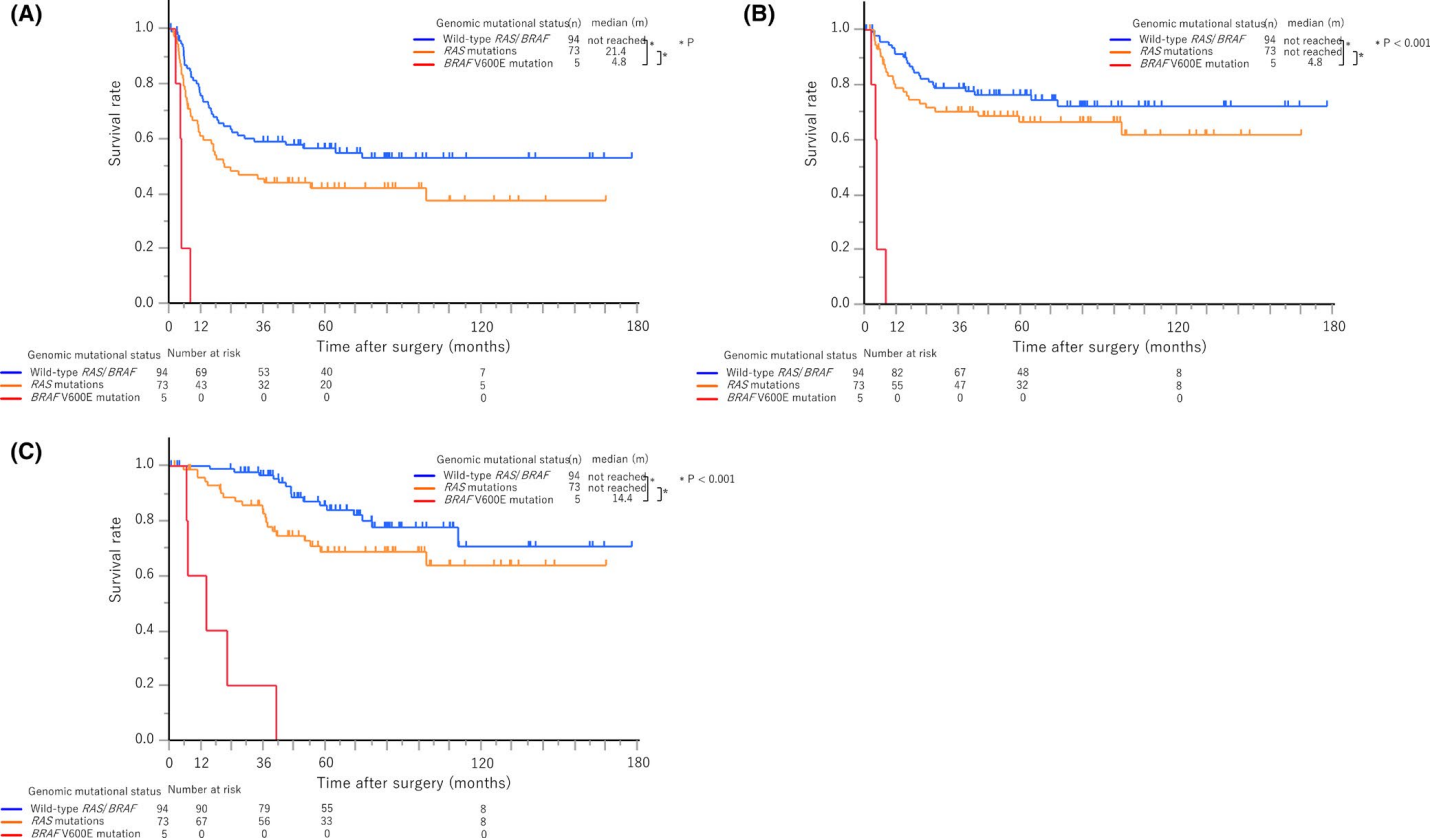
Recurrence‐free survival, time to surgical failure, and overall survival after hepatectomy according to genomic mutational status. A, Recurrence‐free survival. B, Time to surgical failure. C, Overall survival

**FIGURE 3:**
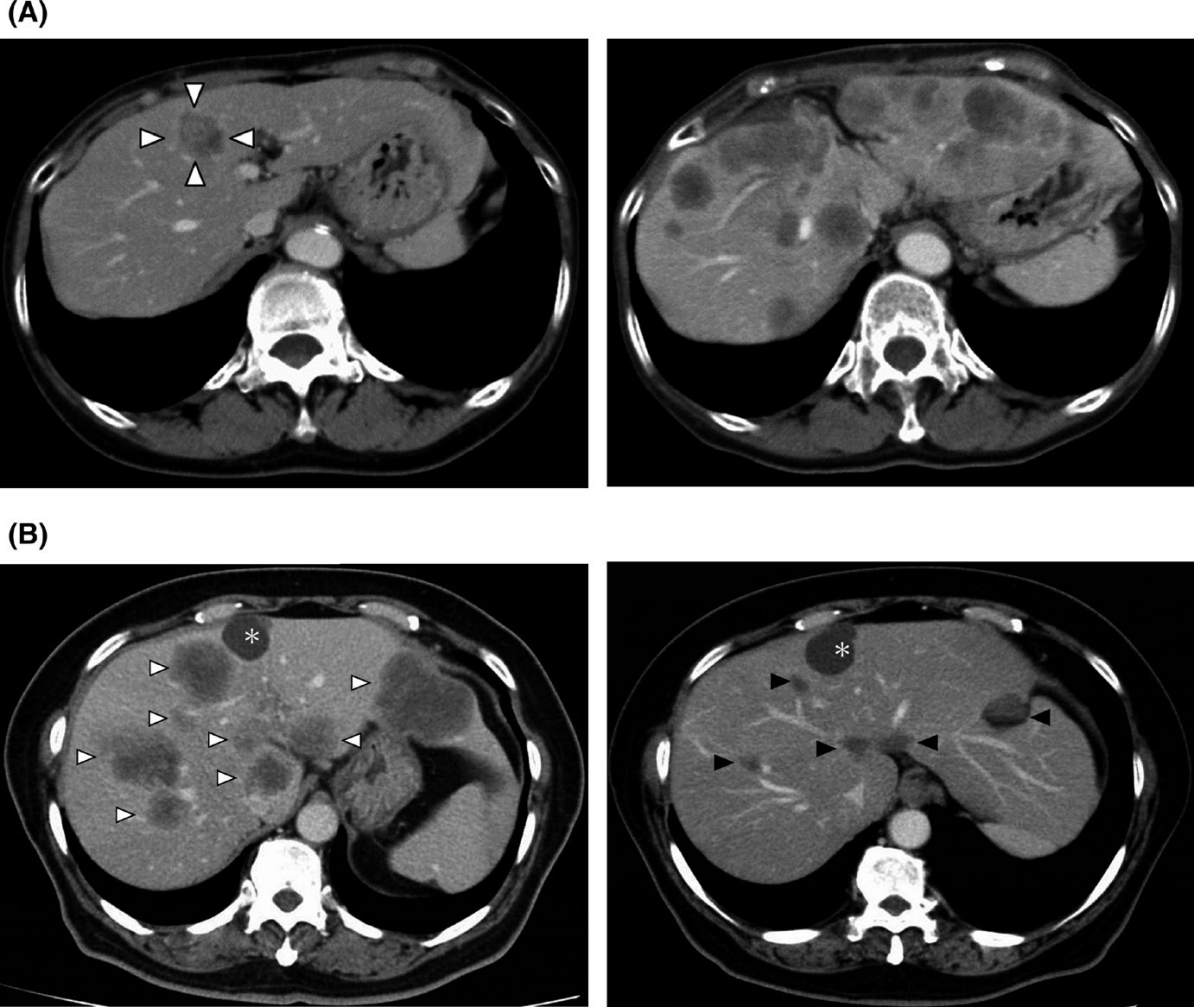
Computed tomography findings of representative patients with BRAF V600E mutation. A, A solitary resectable case. (left) A 71‐year‐old female patient who underwent hepatectomy for metachronous solitary CRLM (triangles). (right) However, the patient developed multiple liver metastases along with lung and peritoneal metastases 4.4 months after hepatectomy and died 6.8 months after surgery. B, An unresectable case. (left) A 61‐year‐old female patient developed metachronous multiple unresectable CRLM (white triangles) and peritoneal metastases along with a liver cyst (asterisk). (right) After receiving FOLFOXIRI plus bevacizumab for 9 months, the CRLM shrunk with good response (black triangles). Afterward, she received the BEACON CRC triplet regimen (encorafenib, binimetinib, and cetuximab) with other treatments and died 36.0 months after the start of the first‐line chemotherapy

The median duration of post‐hepatectomy follow‐up was 72.8 months (range, 0.6–179.2). Recurrences were identified in 5 (100%), 40 (54.8%), and 38 (40.4%) patients among those with m*BRAF*, m*RAS*, and wt*RAS*/*BRAF*, respectively (*p* = 0.01). The median RFS was 4.8 months (95% confidence interval [CI], 2.6–not available [NA]) and 21.4 months (95% CI, 11.9–NA) for m*BRAF* and m*RAS*, respectively. Additionally, the median RFS was not reached (NR) (95% CI, 29.1–NA) in patients with wt*RAS*/*BRAF* (Figure 2). The 1‐year and 3‐year RFS rates were both 0% for patients with m*BRAF*, and 60.9% and 45.4%, respectively, for those with m*RAS*, and 76.7% and 58.9%, respectively, for those with wt*RAS*/*BRAF*. The risk of recurrence was significantly higher in patients with m*BRAF* than those with wt*RAS*/*BRAF* (hazard ratio [HR], 10.9; 95% CI, 4.1–29.0; *p* < 0.001) and m*RAS* (HR, 5.8; 95% CI, 2.1–15.5; *p* < 0.001), and all patients with m*BRAF* developed early systemic unresectable recurrences within 8 months after surgery. A representative case is presented in Figure 3A. Sites of recurrence (liver‐limited vs. systemic) in patients with m*BRAF* (0% vs. 100%), m*RAS* (25.0% vs. 75.0%), and wt*RAS*/*BRAF* (39.5% vs. 60.5%; *p* = 0.14), rates of repeat surgery for recurrences (m*BRAF* vs. m*RAS* vs. wt*RAS*/*BRAF*, 0% vs. 50.0% vs. 52.6%; *p* = 0.08), and rates of systemic chemotherapy for recurrences (m*BRAF* vs. m*RAS* vs. wt*RAS*/*BRAF*, 80.0% vs. 35.0% vs. 42.1%; *p* = 0.15) were not statistically different regardless of the status of the mutations. Regimens of systemic chemotherapy included FOLFOX/CAPOX (oxaliplatin/capecitabine, folinic acid, and fluorouracil, *n* [%], 21 [61.8]), FOLFIRI (irinotecan, folinic acid, and fluorouracil, *n* [%], 10 [29.4]), irinotecan (*n* [%], 2 [5.9]), and UFT plus leucovorin (*n* [%], 1 [2.9]) while target agents were added in 23 patients (67.6%), and rates of each regimen were not statistically different among the three groups (*p* = 0.18). Details of treatment for recurrences are described in the Table S1.

The median TSF was 4.8 months (95% CI, 2.6–NA), not reached (95% CI, 99.0–NA), and not reached (NA–NA) in patients with m*BRAF*, m*RAS*, and wt*RAS*/*BRAF*, respectively. The 1‐year and 3‐year TSF rates were both 0% for patients with m*BRAF*, 78.8% and 70.2%, respectively, for those with m*RAS*, and 91.1% and 78.8%, respectively, for those with wt*RAS*/*BRAF*. TSF was significantly shorter in patients with m*BRAF* than those with wt*RAS*/*BRAF* (HR, 28.7; 95% CI, 9.6–86.1; *p* < 0.001) and m*RAS* (HR, 14.6; 95% CI, 4.8–44.5; *p* < 0.001).

The median OS was 14.4 months (95% CI, 6.7–NA) in patients with m*BRAF*, not reached (95% CI, 99.0–NA) in those with m*RAS*, and not reached (95% CI, NA–NA) in those with wt*RAS*/*BRAF*. The 1‐year, 3‐year, and 5‐year OS rates were 60.0%, 20.0%, and 0%, respectively, for patients with m*BRAF*, 95.8%, 85.7%, and 68.7%, respectively, for those with m*RAS*, and 100%, 96.6%, and 85.5%, respectively, for those with wt*RAS*/*BRAF*. Overall, OS was significantly shorter in patients with m*BRAF* than those with wt*RAS*/*BRAF* (HR, 27.6; 95% CI, 9.5–80.4; *p* < 0.001) and m*RAS* (HR, 9.9; 95% CI, 3.5–27.5; *p* < 0.001). The trend toward poorer survival of m*BRAF* was also consistent irrespective of the sidedness (right or left) of the primary CRC in RFS, TSF, and OS (Figures S1–S3).

Univariable and multivariable analyses for the risk factors of RFS, TSF, and OS are described in Table 2. m*BRAF* was strongly associated with a shorter survival and had the highest HR among all the indicators in terms of RFS (HR: 12.5, 95% CI, 4.3–35.8; *p* < 0.001), TSF (HR: 19.0, 95% CI, 6.1–59.4; *p* < 0.001), and OS (HR: 17.0, 95% CI: 5.2–55.9, *p* < 0.001) in the multivariable analysis.

**TABLE 2:** Univariable and multivariable analyses of risk factors for recurrence‐free survival, time to surgical failure, and overall survival after hepatectomy for solitary resectable colorectal liver metastasis

Characteristics		*n*	Recurrence‐free survival			
			Univariable		Multivariable	
			Median (m) (95% CI)	*p* value	HR (95% CI)	*p* value
**(A) Recurrence‐free survival**						
Patient factors						
Sex	Female	65	23.7 (11.1–NA)	0.33		
	Male	107	64.1 (21.1–NA)			
Age at hepatectomy (years)	<65	70	34.2 (17.1–NA)	0.88		
	≥65	102	74.4 (17.2–NA)			
Primary colorectal tumor factors						
Location	Right‐sided	33	11.9 (5.6–99.0)	0.008	1 [reference]	
	Left‐sided	139	74.4 (24.3–NA)		0.8 (0.4–1.3)	0.28
Depth of invasion	T1–T3	132	36.8 (17.7–NA)	0.69		
	T4	38	51.6 (11.8–NA)			
Lymph node metastases	Absent	72	NR (64.1–NA)	<0.001	1 [reference]	
	Present	99	17.1 (11.7–26.9)		1.9 (1.2–3.1)	0.005
Pathology	Well diff. adenocarcinoma	46	NR (26.9–NA)			
	Others	126	24.3 (16.2–NA)			
CRLM factors						
Timing of CRLM	Synchronous	60	17.1 (10.7–NA)	0.03	1 [reference]	
	Early metachronous <1 year	52	24.3 (11.5–NA)		1.1 (0.7–1.9)	0.66
	Late metachronous ≥1 year	60	NR (45.0–NA)		0.6 (0.4–1.2)	0.15
Diameter of CRLM at diagnosis (mm)	<25.0	83	36.8 (16.3–NA)	0.83		
	≥25.0	89	51.6 (18.3–NA)			
Incidental intraoperative multiple CRLM	Absent	167	45.0 (18.7–NA)	0.53		
	Present	5	NR (5.6–NA)			
Incidental intraoperative peritoneal metastases	Absent	169	54.5 (21.1–NA)	0.03	1 [reference]	
	Present	3	11.8 (4.8–NA)		2.5 (0.7–9.1)	0.15
CEA at diagnosis (ng/ml)	<5.0	65	NR (32.9–NA)	0.03	1 [reference]	
	≥5.0	107	24.3 (16.2–64.1)		1.9 (1.1–3.1)	0.02
CA19‐9 at diagnosis (IU/ml)	<37.0	126	99.0 (23.7–NA)	0.03	1 [reference]	
	≥37.0	46	18.7 (8.2–51.6)		1.1 (0.7–1.8)	0.75
Residual tumor after hepatectomy	R0	163	64.1 (21.0–NA)	0.03	1 [reference]	
	R1	9	16.2 (1.6–NA)		2.1 (0.9–4.6)	0.07
Adjuvant chemotherapy	No	93	32.9 (10.7–NA)	0.28		
	Yes	79	54.5 (21.1–NA)			
Genomic mutational status	Wild‐type *RAS*/*BRAF*	94	NR (29.1–NA)	<0.001	1 [reference]	
	*RAS* mutations	73	21.4 (11.9–NA)		1.5 (1.0–2.3)	0.08
	*BRAF* V600E mutation	5	4.8 (2.6–NA)		12.5 (4.3–35.8)	<0.001

Characteristics		*n*	Time to surgical failure			
			Univariable		Multivariable	
			Median (m) (95% CI)	*p* value	HR (95% CI)	*p* value
**(B) Time to surgical failure**						
Patient factors						
Sex	Female	65	NA (NA–NA)	0.54		
	Male	107	NA (NA–NA)			
Age at hepatectomy (years)	<65	70	NR (NA–NA)	0.94		
	≥65	102	NR (99.0–NA)			
Primary colorectal tumor factors						
Location	Right‐sided	33	99.0 (11.9–NA)	0.12		
	Left‐sided	139	NR (NA–NA)			
Depth of invasion	T1–T3	132	NR (NA–NA)	0.13		
	T4	38	99.0 (42.5–NA)			
Lymph node metastases	Absent	72	NR (NA–NA)	0.002	1 [reference]	
	Present	99	NR (43.9–NA)		2.2 (1.2–4.2)	0.02
Pathology	Well diff. adenocarcinoma	46	NR (NA–NA)	0.32		
	Others	126	NR (NA–NA)			
CRLM factors						
Timing of CRLM	Synchronous	60	NR (42.5–NA)	0.05		
	Early metachronous <1 year	52	NR (74.4–NA)			
	Late metachronous ≥1 year	60	NR (NA–NA)			
Diameter of CRLM at diagnosis (mm)	<25.0	83	NR (NA–NA)	0.21		
	≥25.0	89	NR (99.0–NA)			
Incidental intraoperative multiple CRLM	Absent	167	NR (NA–NA)	0.16		
	Present	5	NR (NA–NA)			
Incidental intraoperative peritoneal metastases	Absent	169	NR (NA–NA)	0.05		
	Present	3	11.8 (4.8–NA)			
CEA at diagnosis (ng/ml)	<5.0	65	NR (NA–NA)	0.09		
	≥5.0	107	NR (99.0–NA)			
CA19‐9 at diagnosis (IU/ml)	<37.0	126	NR (NA–NA)	0.06		
	≥37.0	46	NR (26.5–NA)			
Residual tumor after hepatectomy	R0	163	NR (NA–NA)	0.003	1 [reference]	
	R1	9	23.7 (4.4–NA)		2.6 (1.1–6.4)	0.03
Adjuvant chemotherapy	No	93	NR (NA–NA)	0.92		
	Yes	79	NR (NA–NA)			
Genomic mutational status	Wild‐type *RAS*/*BRAF*	94	NR (NA–NA)	<0.001	1 [reference]	
	*RAS* mutations	73	NR (99.0–NA)		1.4 (0.8–2.5)	0.24
	*BRAF* V600E mutation	5	4.8 (2.6–NA)		19.0 (6.1–59.4)	<0.001

Characteristics		*n*	Overall survival			
			Univariable		Multivariable	
			Median (m) (95% CI)	*p* value	HR (95% CI)	*p* value
**(C) Overall survival**						
Patient factors						
Sex	Female	65	NR (74.4–NA)	0.33		
	Male	107	NR (NA–NA)			
Age at hepatectomy (Years)	<65	70	NR (111.2–NA)	0.83		
	≥65	102	NR (NA–NA)			
Primary colorectal tumor factors						
Location	Right‐sided	33	99.0 (39.9–NA)	0.07		
	Left‐sided	139	NR (NA–NA)			
Depth of invasion	T1–T3	132	NR (NA–NA)	0.05		
	T4	38	99.0 (58.0–NA)			
Lymph node metastases	Absent	72	NR (NA–NA)	0.002	1 [reference]	
	Present	99	NR (78.1–NA)		2.0 (0.9–4.2)	0.07
Pathology	Well diff. adenocarcinoma	46	NR (NA–NA)	0.29		
	Others	126	NR (111.2–NA)			
CRLM factors						
Timing of CRLM	Synchronous	60	NR (111.2–NA)	0.27		
	Early metachronous <1 year	52	NR (78.1–NA)			
	Late metachronous ≥1 year	60	NR (NA–NA)			
Diameter of CRLM at diagnosis (mm)	<25.0	83	NR (NA–NA)	0.19		
	≥25.0	89	NR (99.0–NA)			
Incidental intraoperative multiple CRLM	Absent	167	NR (NA–NA)	0.19		
	Present	5	NR (NA–NA)			
Incidental intraoperative peritoneal metastases	Absent	169	NR (NA–NA)	0.003	1 [reference]	
	Present	3	46.9 (7.3–NA)		8.00 (1.59–40.33)	0.01
CEA at diagnosis (ng/ml)	<5.0	65	NR (111.2–NA)	0.60		
	≥5.0	107	NR (NA–NA)			
CA19‐9 at diagnosis (IU/ml)	<37.0	126	NR (NA–NA)	0.001	1 [reference]	
	≥37.0	46	78.1 (51.6–NA)		2.14 (1.11–4.12)	0.02
Residual tumor after hepatectomy	R0	163	NR (NA–NA)	0.001	1 [reference]	
	R1	9	58.3 (6.7–NA)		5.04 (1.93–13.18)	<0.001
Adjuvant chemotherapy	No	93	NR (NA–NA)	0.94		
	Yes	79	NR (111.2–NA)			
Genomic mutational status	Wild‐type *RAS*/*BRAF*	94	NR (NA–NA)	<0.001	1 [reference]	
	*RAS* mutations	73	NR (99.0–NA)		1.97 (1.02–3.82)	0.04
	*BRAF* V600E mutation	5	14.4 (6.7–NA)		17.02 (5.19–55.85)	<0.001

Abbreviations: CA19‐9, carbohydrate antigen 19–9; CEA, carcinoembryonic antigen; CI, confidence interval; CRLM, colorectal liver metastases; NA, not available; NR, not reached.

### 3.3 Comparison of overall survival between patients with solitary resectable CRLM and those with unresectable CRLM

**FIGURE 4:**
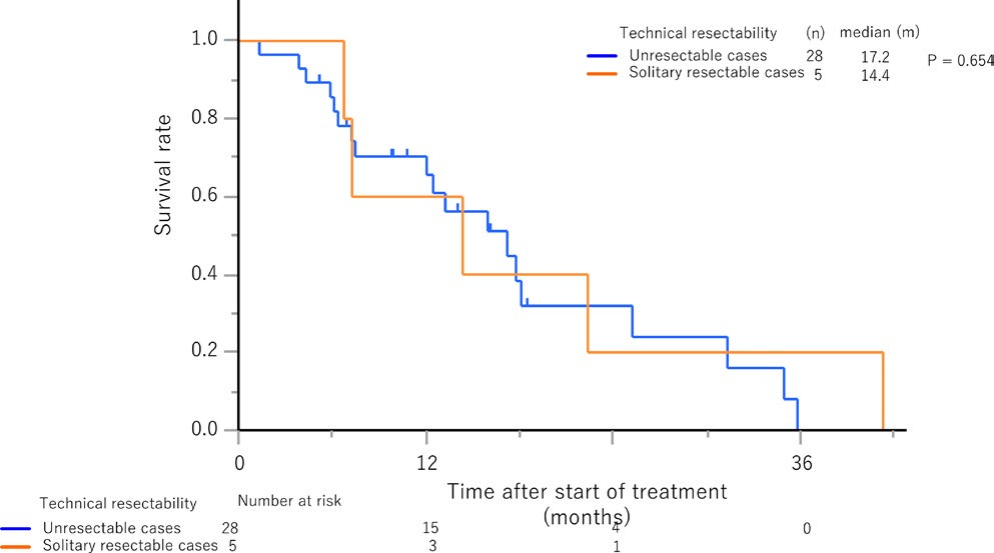
Overall survival of patients with *BRAF* V600E‐mutant colorectal liver metastases depending on the technical resectability

In the second analysis, 28 patients with unresectable *BRAF* V600E‐mutant CRLM who received systemic chemotherapy were identified (Figure S4) and compared with those with solitary resectable *BRAF* V600E‐mutant CRLM identified in the first analysis. A representative case is presented in Figure 3B. Baseline CRLM characteristics were far more advanced in the unresectable group than in the solitary resectable group as reflected in the number of tumors (median, 18 vs. 1, *p* = 0.001) and the rate of concurrent extrahepatic metastases by diagnostic imaging (*n* [%], 20 [71.4] vs. 0 [0]; *p* = 0.005; Table 3). No patients were MSI‐high nor MMR protein‐deficient. FOLFOX/CAPOX (*n* [%], 23 [82.1]), FOLFOXIRI (oxaliplatin, irinotecan, folinic acid, and fluorouracil, *n* [%], 3 [10.7]), and FOLFIRI (*n* [%], 2 [7.1]) were included as first‐line chemotherapy regimens for patients with unresectable CRLM, and target agents were added in 21 patients (75.0%). An anti‐*BRAF* agent (encorafenib) was used in seven patients (25.0%) as second‐ or third‐line chemotherapy as a part of the BEACON CRC trial.^17^ In the solitary resectable group, no patients underwent repeat surgery for recurrences after hepatectomy. FOLFIRI (*n* [%], 2 [40.0]), FOLFOX (*n* [%], 1 [20.0]), and irinotecan (*n* [%], 1 [20.0]) were included as chemotherapy regimens for the recurrences, and target agents were added in two patients (40.0%, Table S1). One patient did not receive any chemotherapy due to rapid deterioration of performance status. No patients received the anti‐*BRAF* agent (encorafenib). The median OS of patients with unresectable *BRAF* V600E‐mutant CRLM was 17.2 months (95% CI, 7.5–25.2), which was nearly identical to that of those with solitary resectable CRLM (HR, 0.78; 95% CI, 0.26–2.33; *p* = 0.65, Figure 4).

**TABLE 3:** Clinicopathological characteristics of patients with *BRAF* V600E mutation according to the technical resectability

Factor	Group	Technical resectability		*p* value
		Unresectable	Solitary resectable	
		*n* = 28	*n* = 5	
Patient factors				
Age at the upfront treatment, median [range]		61 [27, 73]	71 [43, 76]	0.06
Sex, No. (%)	Female	16 (57.1)	2 (40.0)	0.64
	Male	12 (42.9)	3 (60.0)	
Primary colorectal tumor factors				
Location, No. (%)	Right‐sided	17 (60.7)	2 (40.0)	0.63
	Left‐sided	11 (39.3)	3 (60.0)	
Pathology of the primary tumor, No. (%)	Well diff. adenocarcinoma	2 (7.1)	1 (20.0)	0.40
	Others	26 (92.9)	4 (80.0)	
CRLM factors				
Timing of CRLM, No. (%)	Synchronous	24 (85.7)	2 (40.0)	0.05
	Metachronous	4 (14.3)	3 (60.0)	
Number of CRLM at diagnosis^a^, median [range]		18 [1, 102]	1 [1, 1]	0.001
Diameter of CRLM at diagnosis^a^, median [range]	mm	31 [6, 82]	19 [11, 46]	0.24
CEA at diagnosis, median [range]		37.9 [1.3, 5,577.0]	4.0 [3.0, 93.3]	0.13
CA19‐9 at diagnosis, median [range]		986.6 [1.0, 49,740.0]	137.2 [10.2, 563.1]	0.35
Concomitant extrahepatic metastases at diagnosis^a^, No. (%)	Yes	20 (71.4)	0 (0.0)	0.005
	No	8 (28.6)	5 (100.0)	
Extent of extrahepatic metastases at diagnosis^a^, No. (%)	Lung	3 (10.7)	0 (0.0)	
	Peritoneum	11 (39.3)	0 (0.0)	
	Lymph node	10 (35.7)	0 (0.0)	
	Other organs	1 (3.6)	0 (0.0)	
Microsatellite instability	High	0 (0.0)	NA	
Mismatch repair protein	Deficient	NA	0 (0.0)	
Treatment factors				
Initial therapy, No. (%)	Surgery	0 (0.0)	5 (100.0)	
	FOLFOX/CAPOX	23 (82.1)	0 (0.0)	
	FOLFOXIRI	3 (10.7)	0 (0.0)	
	FOLFIRI	2 (7.1)	0 (0.0)	
	Use of target agents	21 (75.0)	0 (0.0)	
Use of anti‐*BRAF* agents in the later therapy, No. (%)	No	21 (75.0)	5 (100.0)	0.56
	Yes	7 (25.0)	0 (0.0)	

Abbreviations: CA19‐9, carbohydrate antigen 19–9; CEA, carcinoembryonic antigen; CRLM, colorectal liver metastases; NA, not available; NR, not reached.

^a^ Those diagnoses were made by pre‐treatment imaging modalities.

## 4 DISCUSSION

In this study, m*BRAF* was associated with intraoperative incidental peritoneal metastases and markedly shorter RFS, TSF, and OS in patients who underwent upfront hepatectomy without neoadjuvant chemotherapy for solitary resectable CRLM. Particularly, m*BRAF* status was the strongest predictor of survival beyond all conventional clinicopathological factors for RFS, TSF, and OS. All patients with m*BRAF* developed early systemic unresectable recurrences within 8 months after surgery. Interestingly, OS after upfront hepatectomy for CRLM associated with m*BRAF* was almost identical to that after systemic chemotherapy for unresectable ones, although baseline tumor characteristics were very different. Inherent tumor biology as assessed by the status of m*BRAF* seemed more important than conventional technical resectability as assessed by radiological findings in judging oncological resectability of CRLM. This study clearly demonstrated the deleterious natural history of patients with m*BRAF* who underwent upfront hepatectomy unselectively from a conventional perspective of technical resectability and elucidated the necessity to detect those with m*BRAF* preoperatively. Thus, CRLM with m*BRAF* can be recognized as oncologically unresectable irrespective of technical resectability, and we propose to perform pre‐treatment genetic testing for m*BRAF* not only for technically unresectable cases but also for resectable ones. To the best of our knowledge, this study is the first to directly compare the survival outcome of patients undergoing hepatectomy for *BRAF* V600E‐mutant CRLM with those treated with systemic chemotherapy for unresectable cases.

Although this study focused on solitary, resectable, and thus, potentially curable CRLM, as opposed to previous studies that included heterogeneous cohorts of patients with variable numbers of metastases and different initial resectability statuses,^9^, ^10^, ^11^, ^12^, ^13^, ^18^ m*BRAF* was consistently associated with elevated levels of baseline serum CA19‐9 and shorter survival after hepatectomy. All but one previous study included patients who received preoperative chemotherapy before hepatectomy, and therefore, the survival outcomes of patients with m*BRAF* in those studies seemed better than they really were because only patients who responded to preoperative chemotherapy favorably were selected for hepatectomy. In contrast, Schirripa et al. reported that RFS and OS after upfront hepatectomy were 5.7 and 22.6 months, respectively.^9^, ^19^ The results of this study were consistent with those of Schirripa et al., and the OS of patients who underwent upfront hepatectomy was nearly identical to that of those with unresectable CRLM treated with systemic chemotherapy. It has been reported that elevated levels of serum CA19‐9 were associated with markedly impaired survival particularly in patients with m*BRAF* compared with those with m*RAS* or wt*RAS*/*BRAF*.^18^ In this study, median level of serum CA19‐9 was elevated to 137.2 IU/ml and it might have augmented the aggressiveness of m*BRAF* compared with m*RAS* or wt*RAS*/*BRAF*. In addition, patient selection criteria of this study that solely included those who underwent upfront hepatectomy unselectively without neoadjuvant chemotherapy must have even enhanced the difference between m*BRAF* and m*RAS* or wt*RAS*/*BRAF*. The biological mechanisms of the increased kinase activity and the elevated levels of CA19‐9 as well as the clinical characteristics of the inclusion criteria elucidated the extremely poor natural history of surgical patients with *BRAF* V600E‐mutant CRLM. It seems that solitary resectable CRLM were just a short process of rapidly progressive systemic disease when complicated with m*BRAF*. The deleterious natural history of patients with m*BRAF* who underwent upfront hepatectomy unselectively from a conventional perspective of technical resectability indicates that CRLM with m*BRAF* can be considered as oncologically unresectable irrespective of technical resectability.

The evidence of upfront hepatectomy for *BRAF* V600E‐mutant CRLM resulting in unexceptionally detrimental outcomes warranted suggesting the proper selection of surgical candidates who my truly benefit from the invasive procedure. Survival outcomes of the previous studies seemed better as the rate of preoperative chemotherapy increases.^9^, ^10^, ^11^, ^12^, ^13^, ^19^ Cremolini C et al. even reported that prognosis of patients with initially unresectable CRLM who responded to chemotherapy (FOLFOXIRI plus bevacizumab) followed by conversion hepatectomy was not influenced by the presence of m*BRAF*, with RFS and OS reaching as long as 11.4 months and unreached, respectively.^19^ We previously demonstrated that systemic chemotherapy should be offered to all surgical candidates with *BRAF* V600E‐mutant CRLM rather than upfront hepatectomy,^12^, ^20^ and this study confirmed that this same strategy should also be applied even for those with solitary resectable CRLM. The recent BEACON CRC trial demonstrated that the novel triplet regimen of encorafenib, binimetinib, and cetuximab, and the doublet one of encorafenib and cetuximab had superior efficacy compared to the standard therapy for patients with previously treated unresectable *BRAF* V600E‐mutant mCRC.^17^, ^21^ Although the efficacy of this triplet regimen is still under investigation in patients with previously untreated *BRAF* V600E‐mutant mCRC,^22^ one potential treatment strategy for technically resectable *BRAF* V600E‐mutant CRLM is the upfront use of this novel triplet regimen followed by hepatectomy in eligible responders.^20^ Therefore, m*BRAF* can be an actionable mutation even for technically resectable CRLM, and we propose to perform pre‐treatment genetic testing for m*BRAF* irrespective of technical resectability.

One potential problem of performing the pre‐treatment m*BRAF* testing might be the relatively low incidence of m*BRAF* among resectable CRLM. The incidence of m*BRAF* among unresectable mCRC was reported around 10% among Western countries and 5% among Asian countries,^23^, ^24^, ^25^, ^26^, ^27^ while it was reported even lower among resectable CRLM. In this study, the incidence among solitary resectable cases was 2.9%. It might be argued that pre‐treatment m*BRAF* testing benefits only a minority of surgical candidates and that surgery should be offered for resectable cases irrespective of m*BRAF* status since it is the standard of care. However, because m*BRAF* is a rapidly progressive disease and accompanies rapid deterioration of performance status with relapse after surgery, not all patients can receive intensive systemic chemotherapy such as the BEACON triplet regimen or FOLFOXIRI plus bevacizumab for relapse after hepatectomy. In fact, one of the five patients who underwent upfront hepatectomy in this study could not receive any chemotherapy after recurrence. By performing pre‐treatment m*BRAF* testing even for resectable cases and providing upfront intensive chemotherapy followed by hepatectomy in responders, we can properly select surgical candidates, maximize the benefit of surgery, prolong the survival time, and might even cure the disease. A recent study regarding circulating tumor DNA (ctDNA) demonstrated that m*BRAF* can be detected before hepatectomy for CRLM.^28^ Since turnaround time for ctDNA was reported as 7 days at median,^29^ pre‐treatment rapid assessment for m*BRAF* status does not delay preoperative surgical management. We believe that m*BRAF* testing opens the door for precision onco‐surgery in resectable CRLM.

This study is limited primarily by its single‐center, retrospective nature with small sample size. Additionally, this study was limited to solitary, resectable CRLM. However, because this study is distinct in its comparison of survival outcomes of patients who were treated with systemic chemotherapy for unresectable CRLM with those who underwent upfront hepatectomy without neoadjuvant chemotherapy, which is not the clinical standard in Western literature,^4^, ^5^ we could shed light on the deleterious natural history of surgical patients with m*BRAF*. Our results convey important guidance to every clinician who takes care of surgical candidates with resectable CRLM. The detrimental impact of m*BRAF* on survival outcomes after surgery warrants its testing before consideration of hepatectomy.

## 5 CONCLUSIONS

Upon complication of technically resectable CRLM with m*BRAF*, survival outcome becomes as poor as those of unresectable cases. Cases with m*BRAF* should be considered as oncologically unresectable. Patients with CRLM should undergo pre‐treatment m*BRAF* testing regardless of technical resectability.

## AUTHOR CONTRIBUTION

Shin Kobayashi, Shinichiro Takahashi, Hiroya Taniguchi, and Takayuki Yoshino: Designed and conceived this study. Shin Kobayashi: Patients enrollment, data acquisition, patient evaluation, and article preparation. Shinichiro Takahashi, Hiroya Taniguchi, and Takayuki Yoshino: Project administration and article review. Masashi Kudo, Motokazu Sugimoto, Masaru Konishi, and Naoto Gotohda: Patient enrollment, evaluation, and article review. Shogo Nomura: Statistical analysis and article review. Motohiro Kojima: Pathological diagnosis and article review. All authors read and approved the final manuscript.

## DISCLOSURE OF COMMERCIAL INTEREST

- Shin Kobayashi, Masashi Kudo, Motokazu Sugimoto, Masaru Konishi, Naoto Gotohda, Shinichiro Takahashi, Hiroya Taniguchi, Motohiro Kojima, and Shogo Nomura have nothing to declare.
- Takayuki Yoshino received research funding from Ono Pharmaceutical Co., Ltd.

## CONFLICT OF INTEREST

The authors declare no conflict of interest.

## ETHICAL APPROVAL

The study conforms to the provisions of the Declaration of Helsinki and was approved by the hospital ethics committee (approval number: 2018‐272).

## Supporting information

Supplementary Material
