# Supplementary material for: BRAF V600E potentially determines “Oncological Resectability” for “Technically Resectable” colorectal liver metastases
Source: Cancer Med. 2021 Sep 18;10(20):6998–7011. doi: 10.1002/cam4.4227 (PMC8525127; doi:10.1002/cam4.4227)
Supplement: Supplementary file 1 — Supplementary Material [file CAM4-10-6998-s001.docx]

**Supporting information**

**Supplemental Table 1 Details of treatment for recurrences according to genomic mutational status.**

|  |  | **Genomic mutational status** | | |  |
| --- | --- | --- | --- | --- | --- |
|  |  | **Wild-type *RAS/BRAF*** | ***RAS* mutations** | ***BRAF* V600E mutation** |  |
| **Factor** | **Group** | **n=94** | **n=73** | **n=5** | **P value** |
| Recurrence, No. (%) | yes | 38 (40.4) | 40 (54.8) | 5 (100.0) | 0.01 |
| Repeat surgery, No. (%) | Yes | 20 (52.6) | 20 (50.0) | 0 (0.0) | 0.08 |
| Systemic chemotherapy, No. (%) | Yes | 16 (42.1) | 14 (35.0) | 4 (80.0) | 0.15 |
| Regimens of chemotherapy, No. (%) | FOLFOX/CAPOX | 12 (75.0) | 8 (57.1) | 1 (25.0) | 0.18 |
|  | FOLFIRI | 3 (18.8) | 5 (35.7) | 2 (50.0) |  |
|  | Irinotecan | 0 (0.0) | 1 (7.1) | 1 (25.0) |  |
|  | UFT plus leucovorin | 1 (6.2) | 0 (0.0) | 0 (0.0) |  |

**Supplemental Figure 1 Recurrence-free survival after hepatectomy according to genomic mutational status stratified by sidedness of the primary colorectal cancer.**

1. **Right-sided**

**
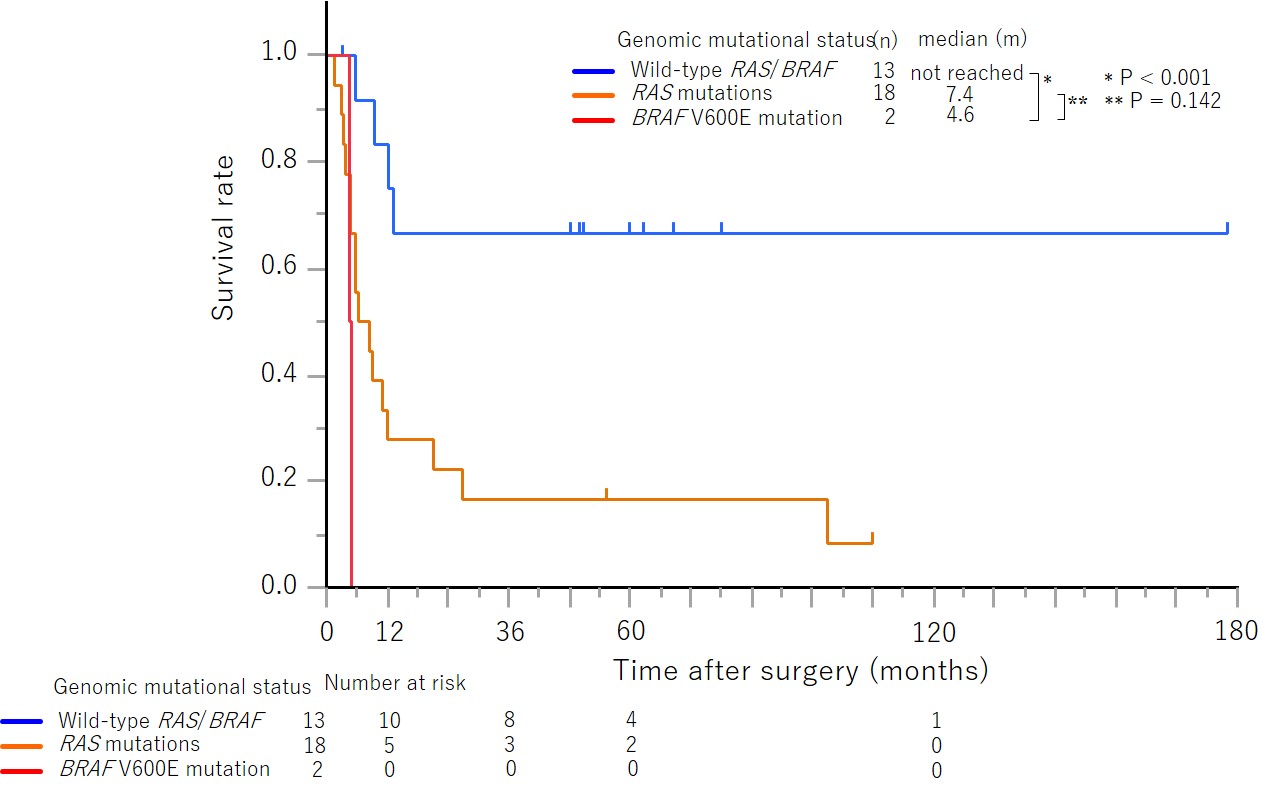
**

1. **Left-sided**

**
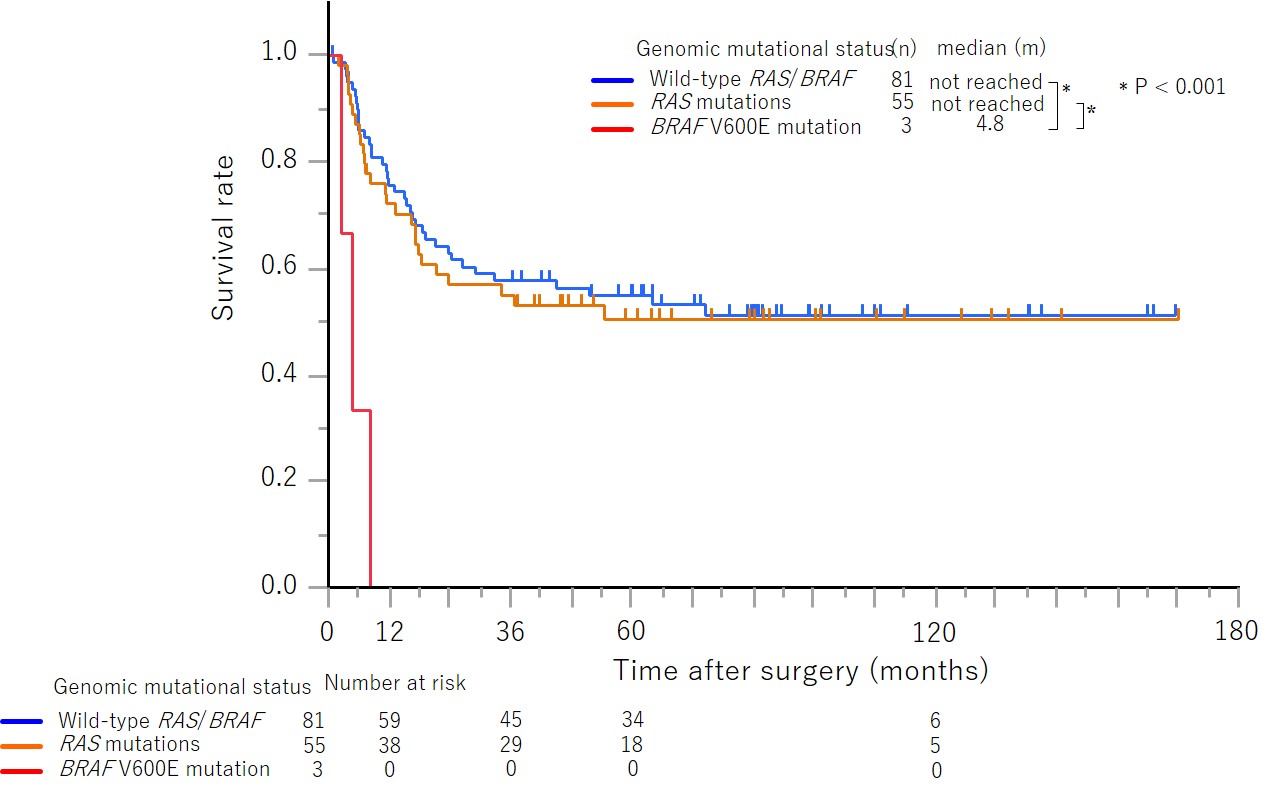
**

**Supplemental Figure 2 Time to surgical failure after hepatectomy according to genomic mutational status stratified by sidedness of the primary colorectal cancer.**

1. **Right-sided**

**
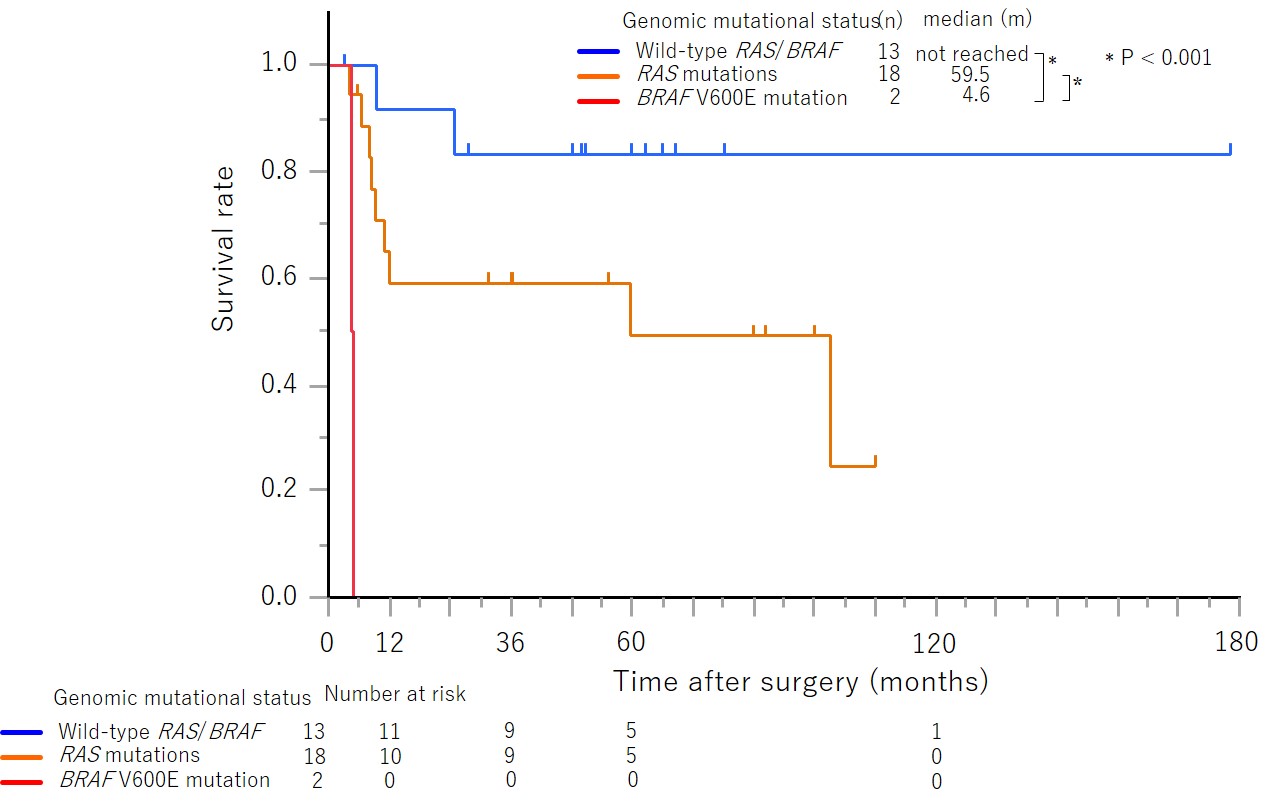
**

1. **Left-sided**

**
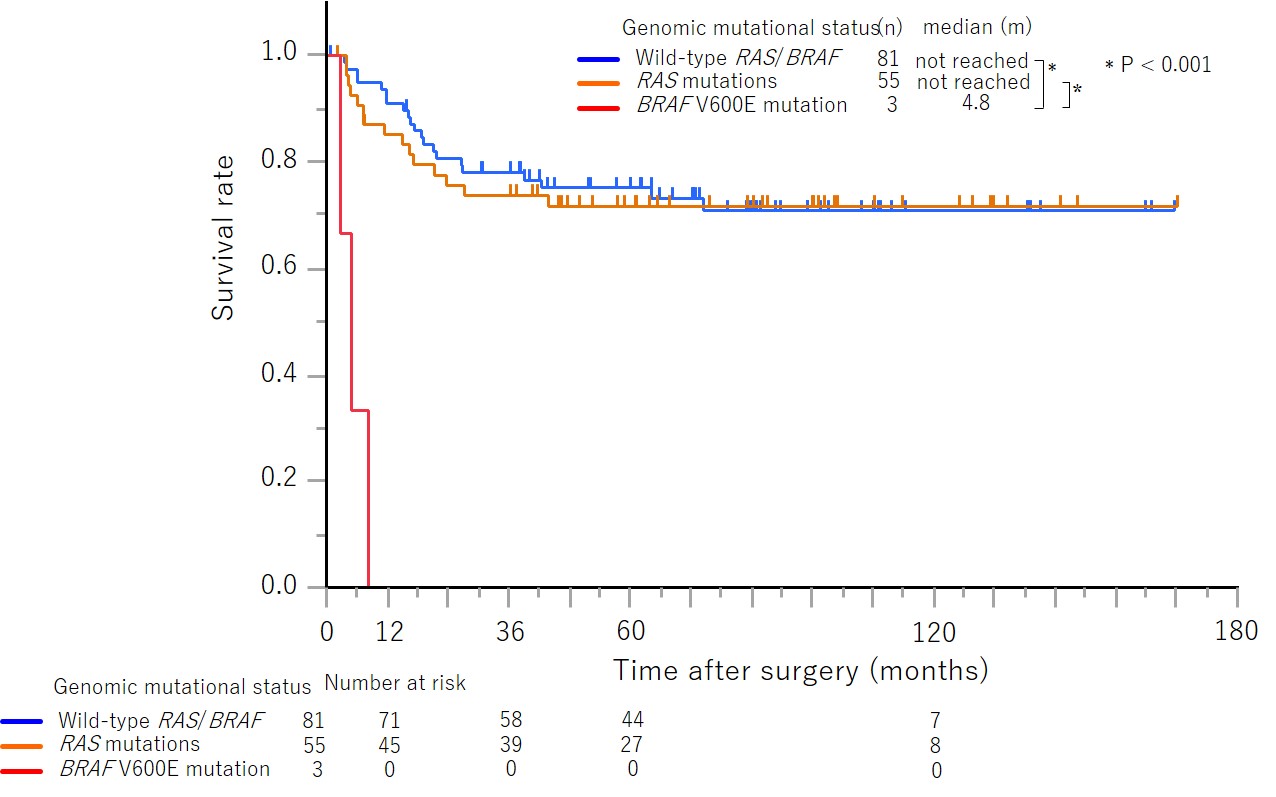
**

**Supplemental Figure 3 Overall survival after hepatectomy according to genomic mutational status stratified by sidedness of the primary colorectal cancer.**

1. **Right-sided**

**
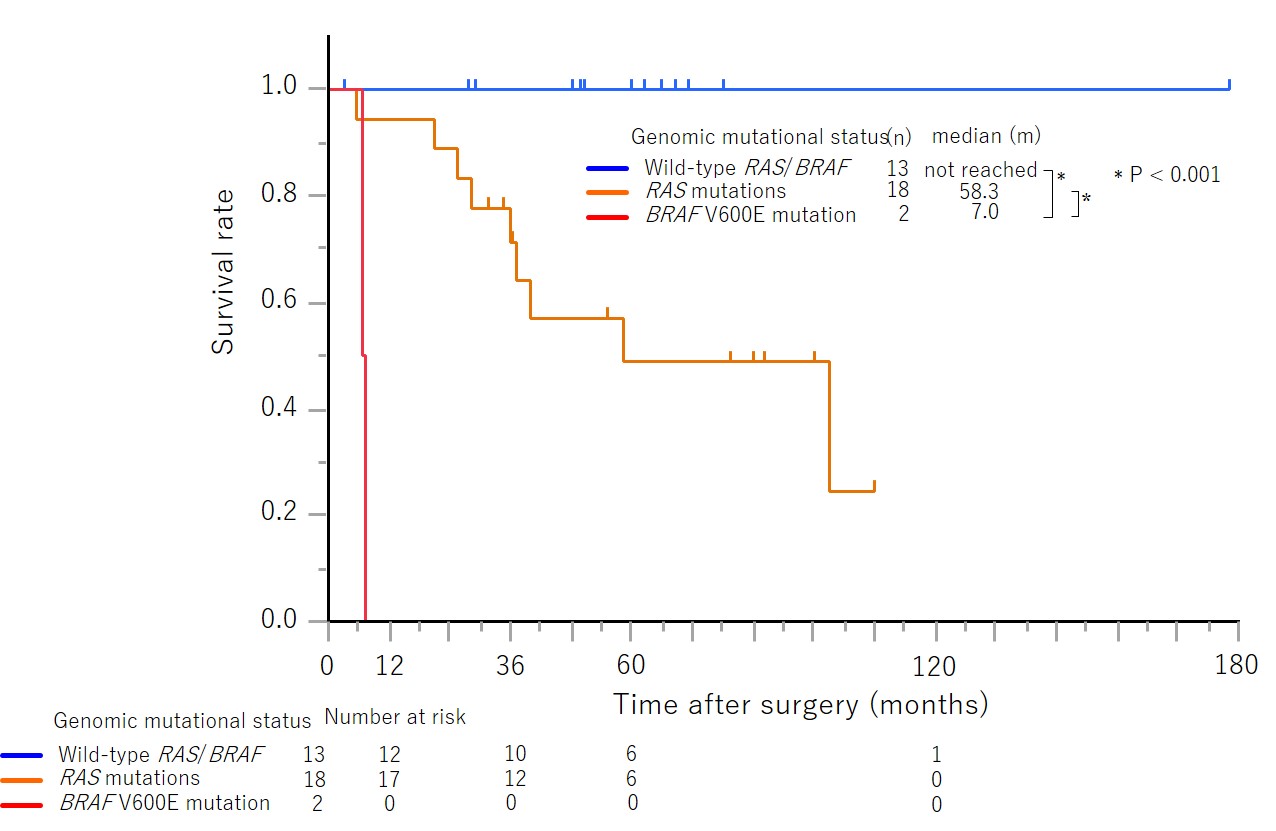
**

1. **Left-sided**

**
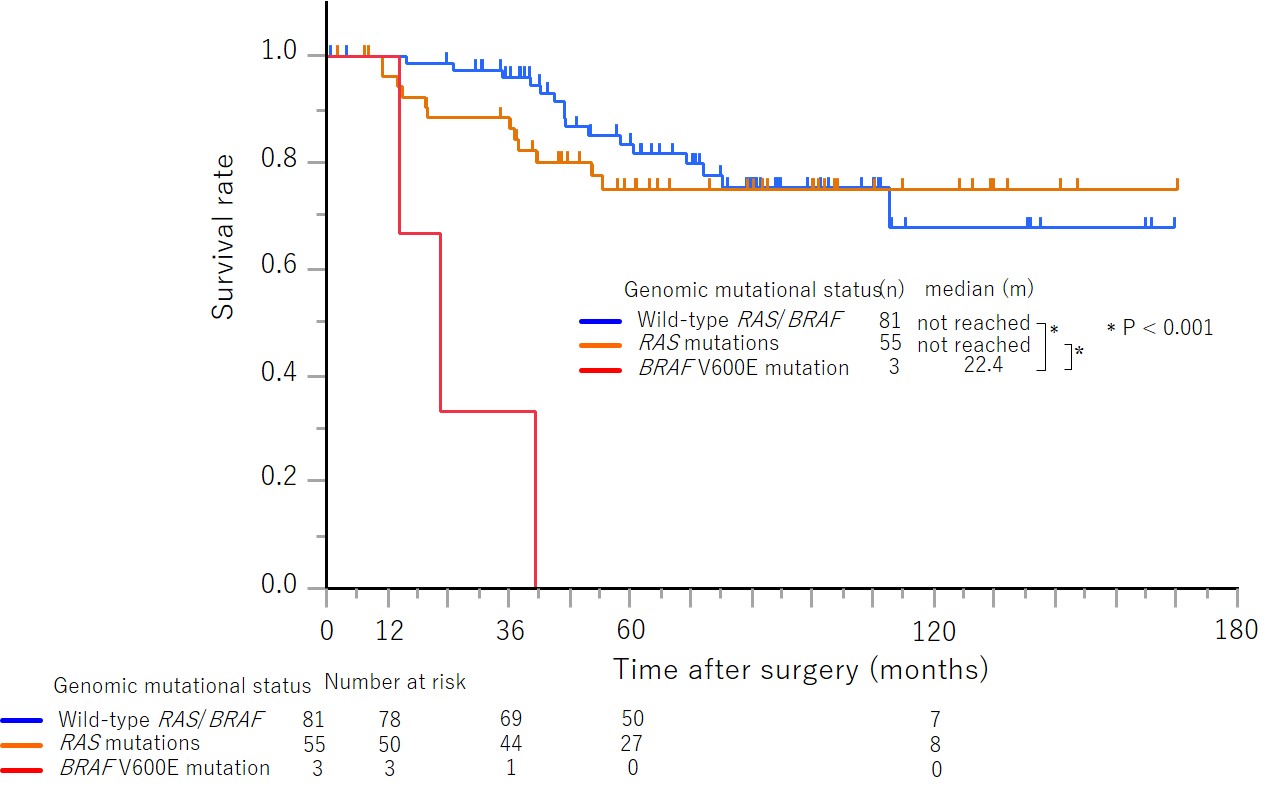
**

**Supplemental Figure 4 Consort diagram of unresectable patients with *BRAF* V600E mutation**

**
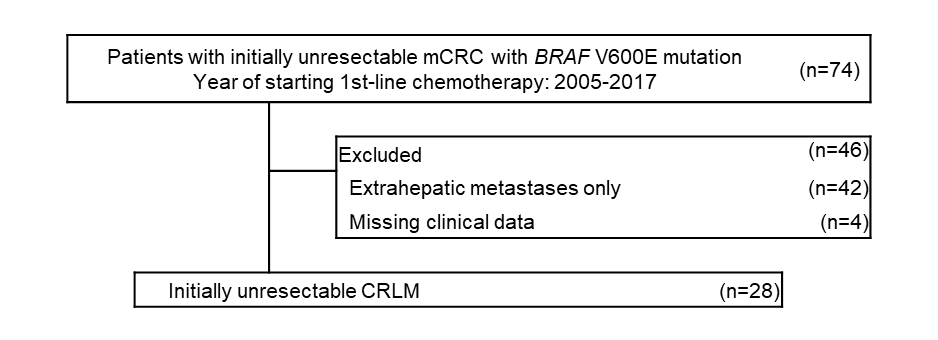
**
